# The Development and Protocol for Testing a Co-Created Digital Intervention (Sentinel) to Improve Mental Well-Being and Help Manage and Prevent Trauma in First Responders

**DOI:** 10.2196/72250

**Published:** 2026-03-18

**Authors:** Nicola Cogan, Alison Kirk, Christoph Graf

**Affiliations:** 1 Department of Psychological Sciences and Health, University of Strathclyde, 40 George Street, Glasgow, G1 1QE, United Kingdom, 44 141 548 ext 4153

**Keywords:** first responders, frontline workers, occupational trauma, digital intervention, participatory methods, mental well-being, trauma management, resilience, co-creation

## Abstract

**Background:**

First responders (FRs) and frontline workers are frequently exposed to traumatic events within their professional roles. This exposure places them at risk of experiencing acute stress, posttraumatic stress disorder, burnout, and other adverse mental health outcomes. Despite growing awareness of these risks, there remains a lack of evidence-based digital interventions (DIs) tailored to meet their unique mental health needs.

**Objective:**

This study aims to address this gap by developing and testing Sentinel, an evidence-based, co-created DI to promote mental well-being, build resilience, and help manage and prevent trauma among FRs and frontline workers. The objectives include exploring their experiences of occupational trauma, identifying their preferences for digital mental health tools, and evaluating the feasibility of the Sentinel intervention.

**Methods:**

The development of Sentinel followed a rigorous, 4-phase approach. In phase 1, we conducted market analysis and in-depth qualitative interviews with 54 FRs from fire services, police, and emergency health sectors. The aim was to explore their mental health needs, barriers to accessing support, and views on the potential role of DIs in addressing these needs. In phase 2, we developed the content for Sentinel by integrating findings from phase 1 with existing evidence, policies, and theoretical frameworks, ensuring the intervention reflected the lived experiences of FRs. In phase 3, a high-fidelity clickable prototype of Sentinel will be tested through co-design workshops, iterative development sprints, and usability evaluations. Feedback from FRs and frontline workers during this phase will refine the app’s design and functionality. Phase 4 involves a mixed methods, nonrandomized feasibility study to evaluate Sentinel’s acceptability, usability, safety, and implementation potential. Quantitative data will be collected from up to 100 FRs, complemented by qualitative interviews with 30 FR participants and 20 health and social care professionals who refer FRs to the intervention.

**Results:**

The co-creation process has proven essential in ensuring Sentinel meets the specific needs of FRs and frontline workers. Preliminary feedback highlights the app’s relevance and usability. Our pilot testing began in August 2025 and is planned for completion by August 2026. As of January 16, 2026, a total of 119 participants have completed the initial survey, and 59 have downloaded the Sentinel app, with 24 having used the app for 6 weeks and completed the follow-up survey. Of this group, 13 participants have consented to an interview. We are on track to meet our target recruitment sample, and these data will provide detailed insights to inform refinements and determine readiness for a larger efficacy trial.

**Conclusions:**

Sentinel represents a novel, personalized digital solution designed to address the unmet needs of FRs and frontline workers exposed to occupational trauma. Future work will evaluate its capacity to improve mental well-being, support trauma recovery, and build resilience.

## Introduction

First responders (FRs), including firefighters, police officers, paramedics, and emergency health care workers, as well as other frontline workers, are at increased risk of experiencing traumatic stress-related conditions resulting from exposure to work-based traumas [1-3]. FRs are exposed to unpredictable and/or dangerous environments, demanding workloads, and heavy emotional labor, making such occupations unique in terms of their demands [4-6]. Almost 90% of FRs describe repeated workplace exposures to incidents that involve direct threats to their lives and/or witnessing the deaths and horrific injuries of others [7,8]. Work-based traumas have been found to have a prolonged negative impact on the mental well-being of FRs, including an increased susceptibility to posttraumatic stress disorder, anxiety, depression, chronic fatigue, insomnia, burnout, and suicide [9-12]. These conditions are associated with higher rates of absenteeism, poorer work productivity, and unhealthy lifestyle behaviors, resulting in poorer physical and mental health [13-15].

The workplace culture of FR organizations strongly esteems strength and self-reliance, which often inhibits FRs from seeking mental health care and support [16]. Seeking help for mental health is often viewed as a form of weakness and/or as a barrier to professional advancement, and more than one-third of FRs experiencing mental ill health do not seek support due to stigmatization, shame, and confidentiality concerns [17-20]. Systematic reviews have synthesized the effectiveness of interventions with FR populations. Smith and Roberts [21] identified 10 intervention studies with FRs and tested their effectiveness, concluding that all interventions lacked quality because of general limitations, such as poor reporting, inadequate sample sizes, low response rates, and sampling bias. A systematic, theory-driven approach to intervention development is recommended to identify modifiable risk factors in at-risk FR groups with prospective studies, followed by the development of interventions to modify core risk factors [22]. The need for a more detailed description of treatment activities or reference to established treatment protocols for interventions with FRs has been recommended [23]. Alden et al [19] found that within 21 intervention studies for FRs, uptake and acceptability of interventions were low, and there was a lack of studies incorporating qualitative data drawn from interviews with FRs to help provide information about which aspects of the intervention were perceived as more or less helpful. Studies included individual or group psychological interventions delivered by clinicians (eg, psychologists) and nonclinicians (eg, experienced supervisors). It was recommended that researchers incorporate FRs’ views on their workplace (eg, stressors and support) and draw participants from multiple organizations to collect both qualitative and quantitative information directly from the FRs [24]. The need for studies that incorporate participatory methods that involve FRs in the early stages of intervention development has also been emphasized [19]; this approach provides insight into FRs’ views on what they would find most helpful in supporting their mental health needs and the circumstances under which mental health interventions work or do not work.

Recent work has highlighted how FRs experience challenges in accessing and engaging in face-to-face mental health care and support, as well as FRs’ anticipation of negative outcomes from treatment, stigma, and how structural barriers inhibit treatment engagement [1]. Intervention research incorporating participatory methods from the onset has been found to improve engagement and uptake [25,26]. Additionally, Winders et al [20] investigated intervention research across FRs and concluded that interventions focusing on the prevention of mental health conditions (as opposed to the treatment) were most effective in reducing the risk of developing such conditions [27]. Although intervention research was limited, many of the systematic reviews agreed that more qualitative research using participatory methods was needed to gauge the specific lived experiences and mental health needs of FRs and that future interventions should aim to address the stigma surrounding mental health help-seeking and prioritize a preventative approach to mental ill health [18-20].

In recent years, digital interventions (DIs) have become popular to overcome existing barriers to accessing mental health support [28,29]. Studies have shown that DIs have been effective in addressing barriers related to stigma and accessibility present within face-to-face interventions [30]. Although DIs in the form of mobile apps or websites have also been shown to present their own problems, as they are dependent on the ability to use and access digital technology [31], they have been shown to be highly effective in the treatment of mental health conditions and have evolved into sophisticated, multifaceted interventions [28,32]. Internet and mobile technologies offer potentially critical ways of delivering mental health support [33]. While there has been very limited research involving DIs with FRs to date, recent studies have reported that DIs have the potential to improve mental health help-seeking behavior among FRs [34,35]. They offer the opportunity to identify early signs of distress and provide timely support, preventing mental health problems from worsening. They can also provide support daily at home or work and at a point when help is most needed and FRs are ready to engage [36]. In addition, they can be implemented on a large scale to meet the increasing demand for mental health support and reduce the burden on mental health services [37]. Nonetheless, research with FRs who are exposed to high rates of occupational trauma is limited, and it remains unclear how DIs can be developed to meet the mental health needs of FRs and ensure that they do not create or exacerbate traumatic experiences [38]. The need for researchers and DI developers to deeply understand users’ needs and/or experiences is increasingly recognized [39]. This requires researchers to directly ask participants about their experiences in a trauma-informed way through interviews, focus groups, surveys, and/or workshops [40]. These forms of participant engagement have the potential to cause retraumatization by requiring people to remember and recount traumatic events [41]. In line with the goal of avoiding retraumatization, researchers should carefully consider how their research may be retraumatizing and work to minimize potential harm. A variety of actions and reflections to mitigate the possibility of retraumatizing research participants are recommended, including understanding the differences between truth-telling and healing, designing more expansive processes of informed consent, developing relational-based interviewing skills, and contextualizing trauma for the populations under study [42]. A trauma-informed approach to DI user research requires researchers to be attuned to the power dynamics in researcher-participant relationships; be transparent about the research’s goals, expectations, and procedure; have skills in managing participant distress; and give the participants autonomy, flexibility, and opportunity to withdraw their participation if needed [40,43]. Under these conditions, having the opportunity to discuss traumatic experiences can have potential therapeutic benefits and can aid the healing process [44]. It is also important that researchers conducting qualitative research on trauma need to recognize the potential for vicarious trauma for themselves and be able to implement appropriate protection and self-care strategies [45], as well as to plan for researcher safety and well-being from the onset of the research [46].

We describe a participatory, co-creation approach to developing a DI (Sentinel) that aims to help manage and prevent traumatic stress among FRs and our plans to pilot test this DI. The importance of understanding and accommodating the perspectives of FRs at all stages of its development and testing is central to our research and design process. The first step involves qualitative research with a wide range of people from the target user populations, carried out at every stage of intervention development, from planning to feasibility testing and implementation [47]. This process enables intervention designers to build a deep understanding of users’ needs in terms of psychosocial factors as well as behavioral elements of the DI [48]. Insights from this co-creation process can be used to anticipate and interpret intervention usage and outcomes and, importantly, to modify the intervention to make it more acceptable, feasible, credible, and relevant to users [49]. Adopting participatory methods that involve FRs in all stages of intervention development can help highlight the distinctive ways that the intervention will address key context-specific social and behavioral issues facing FRs in meeting their mental health needs [28].

The aim of this paper is to describe the development and evaluation of the Sentinel DI, co-created to manage and prevent trauma among FRs and frontline workers. The research is structured into four distinct phases, each with specific objectives:

- Phase 1: To explore the needs, views, and experiences of FRs and frontline workers regarding the use of the Sentinel DI to support mental health. This phase involves conducting in-depth interviews and assessments to gather qualitative data on their mental health challenges, barriers to seeking support, and perceptions of DIs.
- Phase 2: To develop the DI content in consultation with current evidence, policy, theory, and insights gained from FRs’ experiences during phase 1. This collaborative approach ensures that the content is evidence-based, contextually relevant, and tailored to the specific needs of the target population.
- Phase 3: Informed by the outcomes of phases 1 and 2, this phase will involve conducting a series of co-creation workshops and development sprints. The goal is to gather user feedback on a clickable prototype of the DI to test and refine its features, usability, and functionality. Engaging FRs and frontline workers in this iterative process ensures that the DI is user centered and effectively addresses their needs.
- Phase 4: The final phase entails conducting a mixed methods, nonrandomized study to determine the feasibility, acceptability, usability, and safety of the DI. This comprehensive evaluation will inform potential scalability and integration into support systems for FRs and frontline workers.

Given the elevated risk of distress within frontline worker populations, additional safety procedures are in place across both app use and research participation. Sentinel is not a crisis intervention; however, clear signposting to emergency and nonemergency support services is provided within the app and during onboarding. Standardized outcome measures allow for the identification of elevated distress, with participants advised to seek immediate support where risk is indicated. During qualitative interviews, researchers trained in trauma-informed methods will monitor distress, pause or terminate interviews if required, and follow a predefined escalation protocol with senior clinical oversight.

By following this structured, participatory approach, the study aims to develop a DI that is not only effective in managing and preventing trauma but also resonates with and is readily adopted by FRs and frontline workers.

## Methods

### Intervention: Sentinel

In the following phases, we describe the development and protocol for testing a novel digital app, Sentinel, designed to support both trauma management and trauma prevention among FRs. Sentinel distinguishes between trauma management—supporting regulation and coping in response to existing distress—and trauma prevention—reducing the risk of future harm through early identification, psychoeducation, and adaptive support. These functions are delivered through distinct but complementary artificial intelligence (AI)–driven personalization pathways. The innovation lies in the platform’s ability to learn from user interactions and adapt notifications and content over time, enabling the ongoing personalization of interventions that support trauma management and overall health and well-being. The app will be available in a series of sector-specific variants such as Sentinel Green (Health and Social Care), Sentinel Blue (Police), and Sentinel Red (Fire Service), all of which deliver a unique experience specifically designed and tailored to individuals in that sector. Sentinel has been developed through the collaboration of expertise from behavioral science, computer science, physical activity and health, mental health, digital health, clinical psychology, technical product development, and intervention development. This multidisciplinary, combined approach is novel in helping develop an evidence-based and co-created product that is distinct from those currently available in the market.

### Personalization Logic, User Data, and Safety Safeguards

Sentinel uses a transparent, rule-based personalization approach to tailor content while prioritizing safety, fairness, and user autonomy. Personalization is achieved through predefined pathways rather than autonomous or self-learning AI systems. User data informing personalization are limited to nondiagnostic, self-reported, and in-app behavioral inputs provided directly by users. These include onboarding self-report information relating to well-being priorities, user-selected goals, patterns of content engagement (eg, viewed, liked, or skipped content), and optional brief mood check-ins that are used to reassess needs over time.

Decision logic is governed by a rule-based persona framework developed through co-creation with FRs and informed by clinical expertise and evidence-based practice. Personas represent broad, nondiagnostic profiles linked to common patterns of occupational stress rather than clinical conditions. On the basis of user inputs and engagement, Sentinel recommends relevant modules and notifications aligned with stated goals and preferences. No diagnostic inference, risk prediction, or unsupervised algorithmic decision-making is performed. Safety and fairness safeguards are embedded throughout the system. Sentinel does not profile users based on protected characteristics, and conservative thresholds are applied when adapting content to avoid abrupt or inappropriate changes. Sentinel is not a crisis intervention; clear signposting to emergency and nonemergency support services is provided within the app and during onboarding. Human oversight is maintained through multidisciplinary clinical and research governance, with personalization rules refined iteratively through feasibility testing and user feedback.

### Phase 1: User Engagement and Planning

We conducted an initial market analysis with key stakeholders in March 2024, which highlighted the need for an app such as Sentinel in the current digital apps market. Furthermore, we have seen a huge demand for continual professional development workshops (led by NC) on trauma awareness and management from FRs and frontline workers, and this further supports the market gap. We conducted a qualitative investigation into FRs’ exposure to workplace trauma and their views and experiences of using DIs to support their mental health needs. Having gained approval from the University Ethics Committee and informed consent from participants, we conducted in-depth, online one-to-one semistructured interviews with FRs (total, N=54) from a range of organizational contexts (paramedics, n=21; fire and emergency, n=15; emergency health care workers, n=12; and police, n=6) using the Teams platform. All interviewers were provided with in-depth training on conducting qualitative research in trauma, led by a Health & Care Professions Council–registered practitioner consultant clinical psychologist and highly experienced trauma therapist and senior academic. Training included qualitative interview techniques, managing distress, maintaining safety, awareness of vicarious trauma in researchers, and self-care [50]. The training methods used included role-playing, scripted case studies, and online video content on safeguarding, critical incident reporting, and conducting research on sensitive topics [51]. The research team also held regular reflexive meetings to discuss the research process and provide support throughout the conduct of the research. The development of the interview schedule was informed by earlier research [52,53] and focused on FRs’ experiences of exposure to trauma in the workplace and its impact, exploring the domains of (1) mental health and well-being, (2) feelings of safety, (3) help-seeking and supports, and (4) interventions. Questions were developed in accordance with guidelines for ensuring the safety and promotion of resilience of research participants and researchers and were co-created in collaboration with FR organizations during the period of project inception [41,50,54]. Interviews were transcribed in full verbatim and analyzed using a reflexive thematic approach [55]. The major themes developed were (1) the pervasive and salient impact of occupational trauma on mental health (self and others), (2) the demands of the job exacerbating the adverse effects of trauma, (3) insufficient support or access to psychological input following exposure to trauma (lack of psychological safety), (4) stigma and fear of judgment as barriers to mental health help-seeking, and (5) need for accessible, credible, and timely trauma-focused interventions and workplace support (acceptability of a DI). We provide a full description of these themes in our earlier work, and the implications emphasize the importance of implementing a strengths-based, nonpathologizing, and destigmatizing approach to trauma in the workplace as experienced by FRs [2]. The importance of overcoming barriers to accessing mental health support and improving access to evidence-based trauma-focused psychological interventions and workplace support was evident across FRs’ accounts. The perceived acceptability of using a DI to support their mental health needs was reported; however, participants emphasized the need for safety, privacy, and anonymity when using the DI as well as opportunities to connect with other FRs either face-to-face or on a digital platform as important considerations. Participants also reported that they felt that using a DI should be an option as either a stand-alone intervention or in combination with other treatment options (eg, face-to-face therapy, peer support, and online resources). These findings provided evidence of the impact of exposure to workplace trauma on FRs and the importance of overcoming barriers to accessing mental health support. The acceptability of DIs as a means of providing flexible, accessible, and credible help was supported. These themes informed the next phase of intervention development.

### Phase 2: Evidence-Based and User-Informed Content Creation

**Figure 1.**
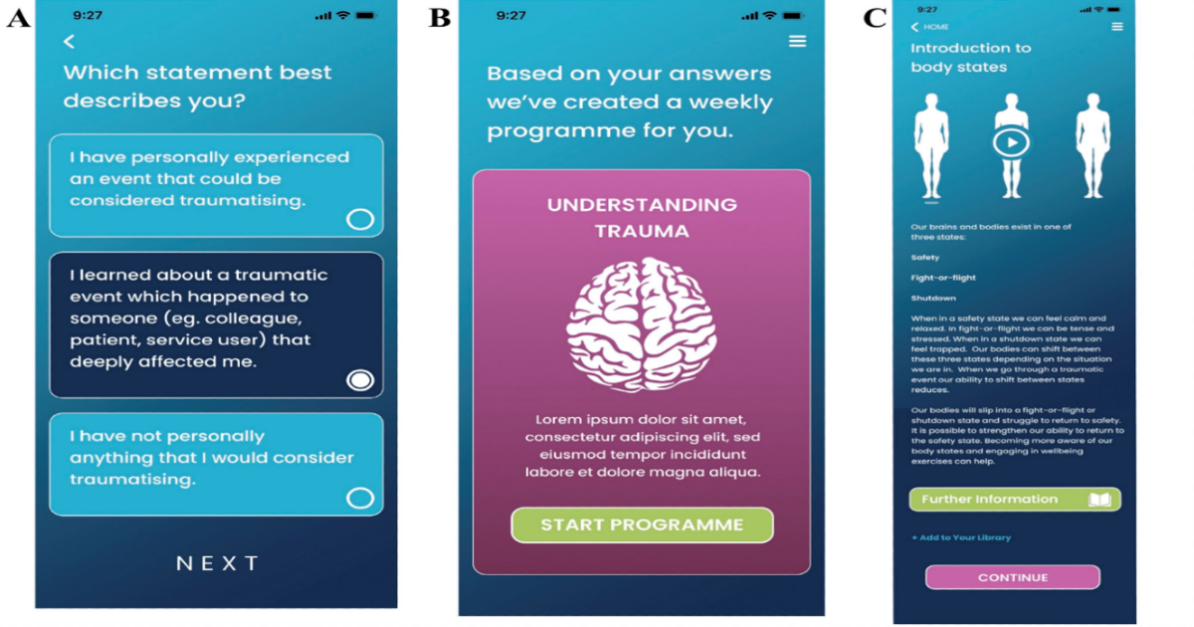
Sentinel Wireframes showing (A) Sentinel asking the user to answer a question to begin determining their “persona*”*; (B) commencement of a tailored program for the user; and (C) one piece of content within the app.

The second phase of developing Sentinel (creation of content) was completed between April 2024 and March 2025. The first step in designing the Sentinel app consisted of developing a list of evidence-based “personas.” These are app user (FR) profiles aligned with information that users input relating to their presenting problems at the onset of using Sentinel (Figure 1A). The “personas” allow Sentinel to decide what intervention modules to give to users, and when, depending on their experiences of traumatic stress and other core presenting problems (comorbid anxiety and/or depressive symptoms; Figure 1B). The second step involved creating the in-app evidence-based module content (Table 1). A knowledge synthesis was adopted to enable the inclusion of a wide range of research to inform content generation and provide a nuanced interpretation and critique of the existing evidence base. The electronic search included the following databases: MEDLINE, PsycINFO, Embase, CINAHL, Scopus, Web of Science, Cochrane Library, and Google Scholar. The following search terms were used: “psychotraumatology,” “trauma,” “first responders,” “healthcare workers,” “mental health,” “help-seeking,” “well-being,” “intervention,” “digital,” and “trauma treatment.” The inclusion criteria were all types of articles, including primary qualitative, quantitative, and mixed methods data; case studies; clinical guidelines; reviews; and opinion texts. The exclusion criteria were articles for which the full text was not available, articles not published in English, or gray literature.

**Table 1.** Categories of Sentinel’s modules*.*

Category	Description	Modules, n	Pieces of content, n
Increasing knowledge of trauma and traumatic stress	Provides psychoeducation on primary and secondary trauma and stress	3	7
Increasing knowledge of posttraumatic growth	Provides psychoeducation on posttraumatic growth and an exercise to foster optimism	3	5
Increasing mind-body and emotional state awareness	Provides exercises to bolster awareness of body state and emotions	12	60
Learning strategies to cope with present distress	Provides self-help and problem-solving strategies to cope with stressors	18	88
Improving general mood and affect	Provides short exercises to improve mood and affect	10	39
Improving sleep	Provides psychoeducation on sleep and strategies to improve sleep	5	16
Improving social connectedness	Provides a strategy to increase and strengthen user’s social connections	1	3
Increasing levels of physical activity	Provides aerobic, strength, endurance, and resistance exercises to promote physical activity	4	67
Reducing levels of sedentary behavior	Provides psychoeducation on sedentary behavior, tips to reduce it, and short exercises	5	9
Increasing resilience	Provides an exercise to increase resilience to future traumatic events	1	3

The articles retrieved (from March 2004 to February 2024) were identified through a search over a 20-year period. In the first round of the search, additional references were identified by a manual search among the cited references. This included evidence-based therapeutic textbooks relevant to the treatment of trauma. With a review of current evidence [27,56,57], policy [58], theory [59-61], clinical guidelines [62,63], and experts by experience in stage 1 (FRs), a total of 297 pieces of content were generated, spread across 62 modules (see Figure 1C for an example piece of content). Module content drew upon cognitive behavioral techniques focused on changing maladaptive thoughts, behaviors, and coping strategies; mindfulness and spirituality to enhance stress management and maintain overall well-being; and approaches to develop skills in physiological regulation and social engagement, informed by Polyvagal Theory [64-67].

**Figure 2.**
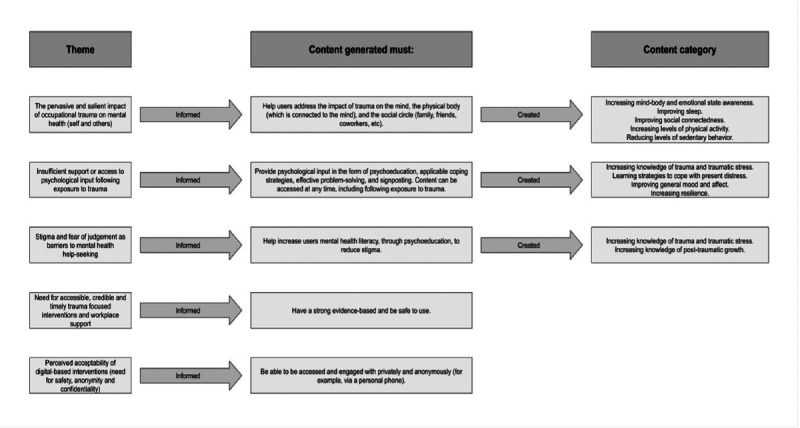
Interview themes informing content generation*.*

Integrating such approaches provides a comprehensive framework for addressing the complex and multifaceted nature of trauma, promoting holistic healing [68]. Sentinel adopts a strengths-based and deshaming approach to dealing with trauma, emphasizing the importance of resilience, positive attributes, and the facilitation of posttraumatic growth [36,69]. The aim is to empower FRs, thereby fostering recovery and personal development beyond trauma [70]. Accessibility rules were set in place to ensure content was clear and easily digestible. Specifically, each piece of content aimed to be no longer than 500 characters and to have good readability. Readability was tested using the Hemingway Editor, where “good” readability was considered a score of 9 or below. In addition, all the content was independently reviewed to check readability, as well as fidelity to the evidence base. A taxonomy was created to describe each module better. This included information on category (what does the module focus on; eg, mental health literacy and coping), type (what does the module specifically focus on within the category; eg, providing psychoeducation and teaching a self-help coping strategy), method (how can the content be engaged with; eg, imagery, writing, using senses, meditation, breathing, and an activity), targets (what “persona(s)” will benefit from the module), symptom (what problem(s) does the module aim to tackle), notification (whether the user receives notifications related to the module), and notification type (what does the notification ask of the user; eg, learn more about a topic and engage in an exercise). The modules are classified into 13 different categories, each informed by the themes gathered in stage 1 (Figure 2). The third step was to design how content would be delivered to the user. Sentinel is a personalized DI, as it will ask users which goals they would like to prioritize (eg, “feel less anxious” or “reduce stress”) and modify content in line with this. It also adapts content according to a number of person-specific characteristics, including work environment and content preferences (ie, what content the user likes or does not like to engage with). Hence, it will take the user’s individual circumstances and preferences into consideration when tailoring its content.

Across the user’s journey, Sentinel will also ask a series of questions to allow it to determine which content users should be given to help deal with common problems associated with traumatic stress. For example, the user may be asked if they experience difficulties falling and staying asleep or recurring nightmares and may be given sleep-related content depending on their answer. The app uses push notifications and reminders to increase and maintain user engagement [71]. These notifications are delivered at regular intervals and are designed to boost FRs’ overall engagement and well-being. Notifications also serve to reassess traumatic stress symptoms and maintain an adaptive, personalized management approach. It is, therefore, possible for a user’s “persona” to change as they use Sentinel.

### Phase 3: User-Informed Prototype Refinement

Informed by the outcomes of phases 1 and 2, our next steps will involve initial testing of the prototype for the Sentinel DI. We will conduct 3 co-creation video call sessions with FRs using Teams and simulations of notice boards or Post-its, and this will be complemented by collective discussions. The time immediately after our co-creation meetings will be dedicated to our development “sprints” to refine the Sentinel app. We have used this process previously in the development of DIs [72,73]. The first co-creation session will present the principles of the DI and then demonstrate and critique a simple initial application prototype. The second co-creation session will review the feedback from meeting 1 and progress during the next design sprint. The third co-creation session will review and finalize the DI design in preparation for the pilot or feasibility study. If a participant cannot attend a group meeting, one-to-one calls will be offered as an alternative. We will recruit participants initially using convenience sampling, followed by snowballing (connections with FR groups and previous survey or interview participants). We will aim to identify 6 to 8 participants for the co-creation groups to maximize the depth of conversation achievable with our discussions [74]. We will aim to include participants representing multistakeholder views across a variety of FR groups, including fire and emergency, paramedics, police, and frontline health and social care workers. Inclusion criteria will include participants aged 18 years and older and interested in contributing to current knowledge of DIs for trauma management in FRs. Ahead of developing the initial minimum viable product of the DI, we created a high-fidelity clickable prototype using a web app mock-up tool (Figma) that is used to gain user insights (phase 3). The prototype allows us to test the basic operation of the DI on various devices with diverse FR user groups (N=100), thereby simulating the user experience of the DI before the intensive app development phase commences [75]. The prototype includes a sample content module, simulated mood reporting and journaling, as well as detailed navigation and functional elements. We have also created an online link to the prototype and a short survey, which will allow us to gather views regarding design, content, and relevance of the DI as well as suggestions for improvements from a larger and more diverse group of potential users.

Co-creation sessions will be guided by structured prompts focusing on perceived relevance and credibility of content, emotional safety, usability and navigation, clarity of language, and appropriateness of personalization and notifications. Participants will be asked what aspects of the prototype feel most and least helpful, whether any content may be distressing or unhelpful, and how the app could better fit the realities of FR work. Feedback will be collated across sessions and reviewed by the multidisciplinary team, with priorities for change agreed upon during postsession debriefs and implemented through iterative development sprints to refine content, functionality, and user flow ahead of pilot testing.

### Phase 4: Pilot and Feasibility Testing of Sentinel

The final phase will involve assessing the feasibility and acceptability of Sentinel and exploring participants’ experiences of using the DI, with quantitative and qualitative data to be collected. Approval to commence with piloting has been obtained from the University Ethics Committee (REF UEC22/92). All data will be stored on secure servers, in line with appropriate security and compliance standards. The data stored are relevant to the functioning of the app and delivery of relevant personalized experiences. This will comply with General Data Protection Regulation and any relevant institutional data governance policies and meet industry standards for information and data security.

### Participants

We aim to pilot test the Sentinel digital app with a target sample of 100 FRs across diverse FR organizations. Participants who report distress associated with exposure to work-related traumas will be recruited from the National Health Service and FR organizations in the United Kingdom. Participants will be recruited through opportunity sampling via online posters distributed within FR organizations, emails sent directly to FR staff members, work intranet bulletin board advertisements, and targeted social media platforms. Interested participants will be eligible if they meet the following criteria: (1) aged 18 years or older, (2) being employed full-time or part-time at the National Health Service and/or an FR organization, (3) understanding the requirements of the study, and (4) having no major mental illness that may impact their capacity to consent to the trial. Given that the focus of the testing phase is to explore the feasibility, acceptability, safety, and usability of the DI, the sample size (N=100) is based on having sufficient variance to examine the feasibility for a larger study across a diverse population [76]. As this study is a feasibility pilot, it is not powered to detect statistically significant pre-post effects. The sample size (N=100) is based on recommendations for feasibility studies to estimate recruitment, retention, acceptability, and variance of outcome measures, which will inform sample size calculations for a future fully powered trial. Quantitative analyses will therefore focus on descriptive statistics, completeness of data, and estimation of effect sizes with CIs rather than hypothesis testing. The nonrandomized design was selected as appropriate for this feasibility study, which prioritizes assessment of acceptability, engagement, usability, and implementation rather than effectiveness. Feasibility will be judged using predefined progression indicators, including successful recruitment within the planned time frame, acceptable retention at follow-up, meaningful engagement with the app over the 6-week period, and satisfactory usability ratings, to inform the design of a future randomized controlled trial. All participants will receive the Sentinel app for 6 weeks. Notifications will encourage FRs to use the app, which is designed to be used in a stand-alone manner alongside routine care.

### Procedure

Participants will first be asked to provide informed consent and then complete an online Qualtrics questionnaire including the trial outcome measures. Following completion of the preintervention online questionnaire, participants will be provided with access to Sentinel and instructed to use the app for a 6-week period. Following the 6-week intervention period, participants will complete an online postintervention questionnaire. As well as looking at differences in pre- and post-intervention outcomes among FRs, the possibility that there might be some variations across different types of FRs with respect to responsiveness to the intervention will be explored. Furthermore, a series of open-ended questions will be asked concerning participants’ views on whether the Sentinel DI had worked for them and how it has impacted their mental health and well-being. An additional subset of FR participants (n=30) will then be asked to take part in an audio-recorded, one-to-one semistructured interview to gain a more in-depth understanding of their experiences of using Sentinel. Furthermore, health care professionals and service managers who referred FRs to the trial (n=20) will be interviewed to explore the acceptability and usability of Sentinel. Participants will be selected according to a sampling framework to capture varied demographics, experiences of work-based trauma, and levels of engagement with the Sentinel app. We will explain to all potential participants that participation is voluntary and that they can withdraw their consent at any point during the study without providing a reason. It will be made clear to FRs that, as the intervention does not replace standard care (but is provided in addition to it), withdrawal from the intervention will not impact their ability to continue to access standard care within the referring service or other sources of support they might access contemporaneously.

All participant data, including sensitive mental health information, are stored on secure, encrypted servers in accordance with General Data Protection Regulation and institutional data governance requirements. Data are pseudonymized at the point of collection, with identifying information stored separately and accessible only to authorized members of the research team. Sentinel does not share individual-level data with employers or third parties, and no identifiable app use, journaling, or symptom data are accessible to organizations. Data are retained only for the duration necessary to meet the study objectives and regulatory requirements, after which they will be securely deleted. Participants are informed of these safeguards during consent to ensure transparency and trust.

### Outcome Measures

To allow a comprehensive evaluation of the effectiveness of the Sentinel digital app in a future randomized controlled trial, we will work with stakeholders and subject experts to determine relevant outcome measures and then test the feasibility of their inclusion within this pilot study. This approach will also enable us to report an indication of effect and provide data to support sample size calculations for future studies. Potential metrics include employee well-being indices, rates of absenteeism and presenteeism, and overall organizational productivity. We will explore the integration of Sentinel with current organizational systems, particularly human resource platforms, which is essential for seamless implementation. We will assess the feasibility of such integration to ensure that Sentinel complements existing processes and enhances user engagement. Understanding the challenges and motivations that organizations face in evaluating the impact of DIs is vital. Factors such as data privacy concerns, resource allocation, and the need for stakeholder buy-in will be considered. By engaging with stakeholders, we aim to identify and address these challenges, ensuring a comprehensive evaluation strategy. This collaborative effort will guide the development of the outcome measures for our pilot trial, ensuring they are relevant, comprehensive, and aligned with organizational priorities. By involving stakeholders in this process, we aim to create a robust framework for assessing Sentinel’s impact, facilitating its successful implementation and adoption within organizations.

As primary outcomes, we will collect information on the total number of participants recruited and retained at different time points, including consent completed, app download, app use over 6 weeks, and follow-up questionnaire completion. We will also collect demographic information, including gender, age, ethnicity, health status, working status, family status, and occupation and recruitment setting. Other proposed psychometric outcome measures to be considered as secondary outcomes include the *Abbreviated Post-Traumatic Stress Disorder Checklist–Civilian* used to measure self-reported trauma symptoms [77]; the *Warwick-Edinburgh Wellbeing Scale* [78]; the *Burnout Measure–Short Version* [79]; the *Depression, Anxiety, and Stress Scale* [80]; the *Neuroception of Psychological Safety* [81]; the *Utrecht Work Engagement Scale* [82]; the *Mobile App Rating Scale* [83]; the *Post-Traumatic Growth Short Form* [84]*;* the *International Physical Activity Questionnaire Short Version* [85]; and the *WHOQOL-BREF* [86]. The validity and reliability of all questionnaires have been well tested [87-89]. More details on the proposed outcome measures have been provided in Table S1 in Multimedia Appendix 1. We also envisage including some physiological outcomes and remote monitoring in a subgroup, and the details of this will be explored during our earlier phases. Following pilot testing, further technology-based adjustments, in addition to delivery components, may require revisions.

### Data Analysis

As this is a nonrandomized feasibility study, analyses will focus on describing recruitment, retention, engagement, usability, and safety outcomes rather than testing intervention effectiveness. Feasibility outcomes will be summarized using descriptive statistics, including counts and proportions for recruitment, retention, app download, completion of follow-up measures, and engagement with Sentinel over the 6-week period, with usability and acceptability measures will be reported using means, SDs, medians, and ranges as appropriate. Exploratory pre-post outcomes will be examined descriptively to inform the design of a future fully powered trial, with standardized mean differences (eg, Cohen *d*) and 95% CIs reported for changes in psychometric measures between baseline and follow-up; no formal hypothesis testing will be undertaken. Missing data will be assessed by reporting the extent and patterns of missingness across measures and time points, with no imputation performed, given the feasibility focus of the study. Exploratory subgroup analyses (eg, by FR role or level of engagement) will be conducted using stratified descriptive summaries only and interpreted cautiously to generate hypotheses and inform future trial design rather than to support inferential comparisons. Qualitative interview data will be analyzed using reflexive thematic analysis, with integration of quantitative and qualitative findings used to inform refinement of the intervention and trial procedures.

### Ethical Considerations

The study received full ethical approval from the University of Strathclyde (Ref UEC22/92 Cogan). Informed consent will be obtained from all participants and their right to privacy explained. The University of Strathclyde was the study sponsor, including management of compensation.

## Results

Our pilot testing began in August 2025 and is planned for completion by August 2026. We anticipate a high attrition rate and will therefore recruit over a full year from several FR organizations using multiple recruitment methods, including organization newsletters and emails, target group presentations, and engagement of key stakeholders within organizations. As of January 16, 2026, a total of 119 participants have completed the initial survey, and 59 have downloaded the Sentinel app, with 24 having used the app for 6 weeks and completed the follow-up survey. Of this group, 13 participants have consented to an interview. We are therefore on track to meet our target recruitment sample.

Once data collection is complete, we expect to report results on the number of eligible FR participants consenting and the total number recruited and retained at different time points, including consent completed, app download, app use over 6 weeks, and follow-up questionnaire completion. We will also report on information about recruitment setting to inform the sampling strategy of a future randomized trial; completeness of outcome measures, including psychometric questionnaires and an indication of effect; attrition rates and reasons for withdrawal; the range of FR organizations offered Sentinel; participant completion rates for Sentinel; Sentinel platform use and engagement analytics; and safety of the Sentinel app and our trial procedures. We also expect to report on perceived barriers and enablers to integration and uptake into existing mental health care provider pathways. Exploratory analyses will be conducted to examine potential variation in intervention responsiveness across different FR groups. Given the feasibility focus and anticipated sample sizes within subgroups, these analyses will be descriptive and exploratory in nature, using stratified summaries and estimation of pre-post change scores by responder group rather than formal hypothesis testing. Findings will be used to inform subgroup specification and analytic strategies for a future fully powered trial.

## Discussion

### Implication of Work

In this paper, first, we have described the process for the development of the Sentinel DI adopting a participatory co-creation approach, and, second, the protocol for testing the DI. We outlined the phase-based approach we have adhered to in developing the DI and our protocol for ascertaining the feasibility, acceptability, safety, and usability of the DI and how best to integrate it into existing routine care pathways for FRs. As recommended for DI development, we have emphasized the importance of FR user engagement and planning during all phases of the process [90]. To the best of our knowledge, this is the first DI of this type to be co-created with FRs worldwide. We have carried out in-depth qualitative interviews with FRs, held regular advisory group meetings with key stakeholders, incorporated input and feedback from this work into the development of the DI content and user interface, and created evidence-based content for the DI [2]. Ahead of developing the minimum viable product of the DI, we created a high-fidelity clickable prototype that will be used to gain user insights before further app development and pilot testing commences. The user-driven, co-creation approach we have adopted is intended to ensure that the DI is engaging and relevant and reflects the needs and experiences of FRs with a focus on trauma management and prevention, deshaming mental health help-seeking, and fostering resilience and posttraumatic growth [36,91].

The DI has the potential to provide many benefits, including increased awareness of trauma and trauma-related symptoms; accurate identification of primary and secondary trauma; immediate support for FRs and emergency frontline workers; reduced costs and waiting times for therapy; early-stage trauma management and prevention; remote triage leading to self-management; advice on seeking in-person treatment; advanced-stage signposting to mental health professionals; and a unique and tailored experience based on sector, trauma type, and data science [92]. Technology is leading to rapid change in the way mental health interventions are increasingly being delivered. To create inclusive opportunities for people who experience mental health challenges associated with trauma, any DI development needs to not only address the present time but also to anticipate and influence future technological directions [93]. To prevent current inequalities and biases from becoming “hardwired” into DIs and future advancements in algorithms for AI, it is essential that people with lived experiences of mental health challenges are involved in the co-creation of such technologies [94]. People with mental health challenges are one of several groups that are more likely to be digitally excluded (do not have access to or are unable to access the internet). Therefore, they may not have the opportunity to engage in DIs designed to support their mental health needs and experience further exclusion [91]. Developments in technology to help support and treat people experiencing trauma are raising new ethical questions about the current and future rights of people accessing DIs, in particular their rights regarding surveillance, privacy, and what it means to be human [95]. In our work with Sentinel, we aim to consider such broader ethical and moral issues and continue strengthening our partnership working with FRs and other key stakeholders in our co-creation process and future AI innovations [94]. This includes ensuring privacy and confidentiality, addressing potential biases in algorithms, and ensuring that DIs complement, rather than replace, human care [96]. Training and education for mental health professionals and community organizations on the use of DIs are crucial [97]. This ensures that they are equipped to integrate these tools into their mental health practices and can provide support to individuals using such interventions. Training should also emphasize the importance of maintaining a human-centered approach to mental health care [98].

### Limitations

As this protocol is for a pilot study, we are unable to conclude whether the Sentinel intervention will bring about any clinically significant change after intervention. However, this study design, with its focus on feasibility, acceptability, safety, and usability, will provide valuable insights that will inform parameters for a future randomized trial, should this be indicated. The inclusion of organizationally led impact outcomes, psychometric measures, and open-ended questions asking FR users whether the DI worked for them and how it had impacted their mental health and well-being will provide some preliminary evidence of its efficacy. Moreover, feedback from multiple stakeholders, including those who refer FRs to the feasibility trial, will allow for improvements in the Sentinel app, trial procedures, and plans for implementation. In our work, to date, we have not yet engaged with military FRs and/or family members of FRs; we would view this as a priority in our next steps in DI development, given their central role in providing social and/or familial support to FRs [99]. This aligns with emerging work on persistent traumatic stress exposure [100], which highlights the importance of considering ongoing and cumulative trauma within relational and support systems.

### Conclusions

In conclusion, the co-creation process has been instrumental in developing Sentinel, a personalized digital app tailored to the unique needs of FRs. This participatory approach has ensured that the intervention is both relevant and user-centered. However, further research is essential to evaluate Sentinel’s acceptability, usability, feasibility, and safety across diverse FR service settings. Such evaluations will inform necessary refinements, maximizing the app’s effectiveness in trauma management and prevention. Addressing potential concerns and safety issues is crucial to enhance user engagement and ensure the intervention’s success. This forthcoming feasibility trial represents a pivotal step in bolstering support for FRs, highlighting a critical area for both research and intervention development. Pilot testing of our DI lays the groundwork for a more definitive solution that can be rigorously tested in future studies and ultimately implemented to support FRs across various service environments.

## Supplementary material

10.2196/72250 Multimedia Appendix 1 Details of proposed outcomes measures.

## Abbreviations

AI: artificial intelligence
DI: digital intervention
FR: first responder
