# Supplementary material for: The Development and Protocol for Testing a Co-Created Digital Intervention (Sentinel) to Improve Mental Well-Being and Help Manage and Prevent Trauma in First Responders
Source: JMIR Res Protoc. 2026 Mar 18;15:e72250. doi: 10.2196/72250 (PMC12998612; doi:10.2196/72250)
Supplement: Multimedia Appendix 1 [file resprot-v15-e72250-s001.docx]

Multimedia Appendix 1

Supplementary Table 1: Details of proposed outcomes measures

| Outcome measures | Description |
| --- | --- |
| Demographics | Gender, age, ethnicity, health status, working status, family members, and occupation. |
| *Other proposed psychometric outcome measures* | |
| *Abbreviated Post-Traumatic Stress Disorder Checklist – Civilian* (APCL-C)    (Lang & Stein, 2005). | To measure self-reported trauma symptoms. The APCL-C provides assessment for difficulties associated with post-traumatic stress in the civilian population. The 6-item APCL-C presents participants with common complaints in response to stressful life experiences and asks how frequently they have been bothered by each in the past month. Items include ‘Repeated, disturbing memories, thoughts, or images of a stressful experience from the past’ and ‘Feeling irritable or having angry outbursts’. Responses are given on a 5-item Likert scale ranging from 1 (not at all) to 5 (extremely); here, higher scores are suggestive of difficulties with post-traumatic, acute stress which requires further professional attention. The measure is reported to have sound psychometric properties (McCutchan et al., 2016). |
| *Warwick-Edinburgh Wellbeing Scale (WEMWBS):*    (Tennant et al., 2007) | To measure mental wellbeing. This scale has 14 items and is scored on 5-point scales (*1 = None of the time – 5 = All of the time)*, where higher scores indicate higher levels of mental wellbeing. The WEMWBS has demonstrated both high internal consistency and high test-retest reliability (Haver et al., 2015). |
| *Burnout Measure – Short Version (BMS-SV):*  (Malach-Pines, 2005) | A 10-item scale designed to measure burnout across diverse occupational and community contexts. Participants will be asked how often they feel, for example, ‘Tired’, ‘Worthless/Like a failure’, ‘I’ve had it’. Responses are given on a 7-point scale ranging from 1 (never) to 7 (always), where higher scores are associated with higher levels of physical, emotional, and mental burnout. |
| *Depression, Anxiety, and Stress Scale (DASS-21)*    (Lovibond & Lovibond, 1995) | To measure depression, anxiety and stress. It is a 21-item measure that is scored via 4-point scales (*0 = Did not apply at all – 3 = Applied to me very much, or most of the time)*, whereby higher scores reflect higher levels of distress. This measure calculates scores for depression, anxiety, and stress. Previous research has shown the DASS-21 to have good psychometric properties (Brown et al., 1997). |
| *Neuroception of Psychological Safety* (NPSS)    (Morton et al., 2022) | A 29-item self-report measure that has three sub-scales (social engagement, compassion, and body sensations) and asks participants to rate how well statements describe their feelings over the past week. Statements include ‘I felt valued’, ‘I felt compassion towards others’ and ‘My heartrate felt steady’ and responses are given on a 5-point Likert scale ranging from 1 (strongly disagree) to 5 (strongly agree). Higher scores on the NPSS indicate higher levels of psychological safety at the individual level. This measure has documented sound psychometric properties (Cogan et al., 2024b) |
| *Utrecht Work Engagement Scale (UWES)*    (Schaufeli, et al., 2002) | To assess the extent to which participants report feeling positive, fulfilled, and in a work-related state of mind, characterized by vigour, dedication, and absorption. It is a 17-item measure scored via 7-point scales (*0 = Never – 6 = Always*), whereby higher scores exhibit higher levels of work engagement. The UWES calculates a total score plus 3 subscale scores, including: vigour (i.e., high levels of energy, persistence, and resilience towards one’s work activities), dedication (i.e., high levels of enthusiasm and investment in one’s work, with a sense that it has meaning and purpose), and absorption (i.e., being fully and happily engrossed in one’s work). This scale has documented high psychometric properties (Schaufeli, et al., 2002). |
| *Mobile App Rating Scale (MARS)*    (Stoyanov, et al., 2015) | To classify and assess the quality of mobile health (mHealth) apps. The MARS has shown to be a reliable (Terhorst et al., 2020) and widely applied (Davalbhakta et al., 2020; Salazar et al., 2018) way to systematize assessment of the quality of mobile apps. The MARS includes 23 items grouped in different sections: engagement, functionality, aesthetics, information quality, and subjective quality. |
| *Post-Traumatic Growth Short Form (PTG-SF)*    (Cann et al., 2010). | A measure of post-traumatic growth that includes 10 items on a six-point Likert scale, where a score of zero indicates ‘not at all’ and a score of five indicates ‘very much’; for example, ‘I discovered that I am stronger than I thought I was’. This measure has been found to have good internal consistency and reliability (Leykinet et al., 2013). |
| *International Physical Activity Questionnaire short version* (IPAQ-SV)    (Craig et al., 2003) | To collect information on physical activity and sedentary activity. This 7-item questionnaire assesses the types and intensity of physical activity and sitting time that people do as part of their daily lives. It has been widely tested and is designed to be used by adults aged 18–65 years (Craig et al., 2003). |
| *World Health Organization Quality of Life Instrument (WHOQOL-BREF)*  Skevington et al., (2004) | Developed by the WHO, it assesses quality of life in four domains: physical health, psychological wellbeing, social relationships, and environmental factors. It is highly regarded for its global applicability and focus on subjective wellbeing. |
